# Clinical Management of Two Root Resorption Cases in Endodontic Practice

**DOI:** 10.1155/2016/9075363

**Published:** 2016-08-28

**Authors:** Jozef Mincik, Daniel Urban, Silvia Timkova

**Affiliations:** ^1^Private Dental Practice, Vystavby 3, 040 11 Kosice, Slovakia; ^2^Mint Dental, Private Dental Practice, Ostravska 8, 040 11 Kosice, Slovakia; ^3^Faculty of Medicine, Department of Dentistry and Maxillofacial Surgery, Pavol Jozef Safarik University, Rastislavova 43, 040 01 Kosice, Slovakia

## Abstract

Root resorption is a pathological process involving loss of hard dental tissues. It may occur as a consequence of dental trauma, orthodontic treatment, and bleaching, and occasionally it accompanies periodontal disease. Although the mechanism of resorption process is examined in detail, its etiology is not fully understood. Wide open apical foramen is more difficult to manage and the root canal may often overfill. In this report we present two cases of root resorption and describe means for its clinical management. We conclude that useful measure of a success or failure in managing root resorption is the persistence of the resorption process. It is a clear sign of an active ongoing inflammatory process and shows the clinical need for retreatment.

## 1. Introduction

In healthy organism, the outer and inner walls of dental root are protected by a thin antiresorption barrier. A layer of precementum protects the outer wall while predentin and odontoblasts protect the inner wall of root dentin. Resorption cells can under no conditions colonize nonmineralized surface [1, 2]. It has been long established that multiple factors, mechanical, chemical, or thermal, can cause premature mineralization of protective barriers and initiate the process of resorption [3]. The transformation of precursors into clastic cells is induced by cytokines, of which interleukin-1*β* plays crucial role [1, 4]. More recent studies investigate the role of extracellular matrix components such as collagen type I, fibronectin, and osteoponin taking part in regenerative process of resorption lesions [5].

Root resorption is a very common finding. This has been well established for some time in the works of Harvey and Zander [6] and Massler and Malone [7]. In a more recent study Tsesis et al. [8] investigated the prevalence of root resorption in Middle Eastern population, finding that almost 29% of teeth were affected. According to this study, the most common type of resorption was related to pulpal infection. External root resorption can also be caused by an injury, either sudden (trauma, replantation) or persistent over time (excessive orthodontic force, impacted teeth, tumors, and cysts) [9, 10]. Holan et al. also investigated and classified rather atypical external root resorptions and associated them with trauma [11]. Some cases of external resorption can be classified as idiopathic with unknown or unproven causality. It occurs as a solitary or multiple form. Hyperparathyroidism, hypocalcaemia, hypophosphatemia, and Paget's disease may play a role in the development of these lesions [12–15]. External root resorption often manifests itself radiographically as shortened root in the apical area. Internal root resorption originates in the inner wall of the root canal system. Radiograph often reveals well described radiolucency along the root canal and/or the coronal section of the pulp. Central incisors are the most frequently affected teeth; this can be explained by the fact that they are the most vulnerable teeth to accidents and injuries. Even a minor posttraumatic hemorrhage can develop into a resorption granuloma [16].

Root resorption treatment is directly related to the causative factor. External periapical inflammatory root resorption (see Case 1) and internal root resorption (see Case 2) are caused by pulpal infection [17]. The adequate root canal treatment will provide sufficient control of bacteria and hence cease the resorption process. Root resorption, being a progressive condition, calls for immediate endodontic intervention. Tronstad [18] advocated the use of calcium hydroxide as a temporary intracanal medicament in the management of root resorption. According to the author, the high alkaline pH will neutralize the lactic acid secreted by osteoclasts and the demineralization process will cease. The calcium hydroxide treatment is discontinued when a continuous periodontal ligament space becomes visible radiographically. This process may take up to 6–12 months [19]. Thermoplastic gutta-percha is recommended for a permanent filling. Proper three-dimensional obturation of the root canal provides satisfactory seal as it can also be condensed into the undercutting areas of an internal resorption lacuna [20].

## 2. Case Reports

### 2.1. Case 1

**Figure 1:**
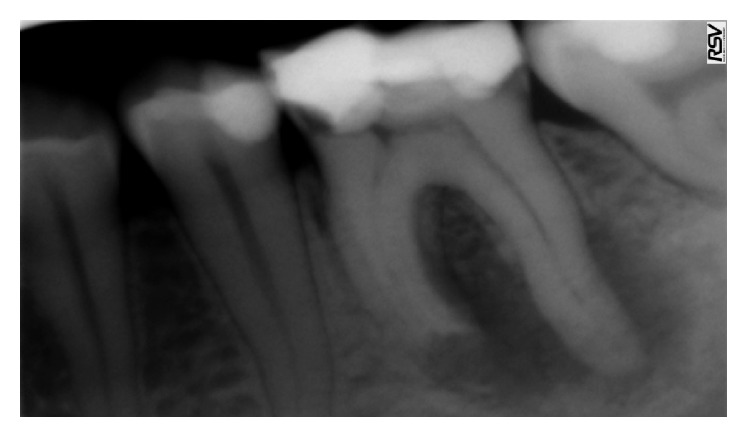
Initial radiograph.

**Figure 2:**
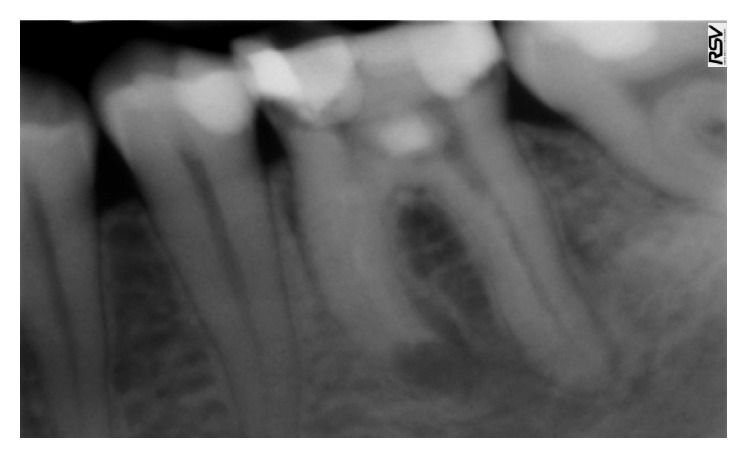
Radiograph with temporary therapeutic agent.

**Figure 3:**
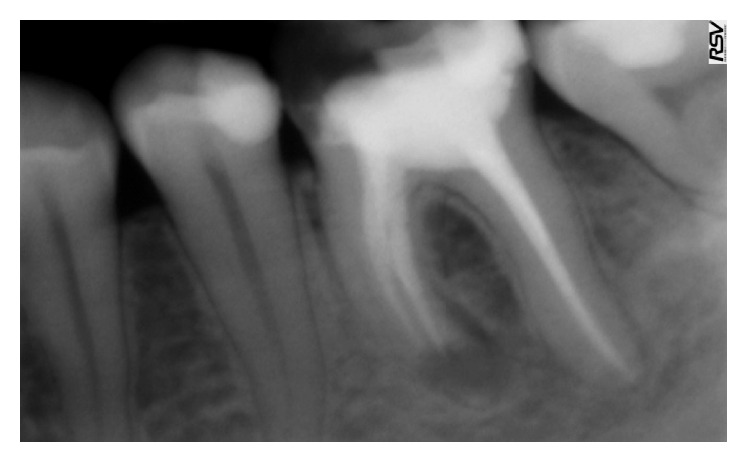
Radiograph at 2-month follow-up.

A 34-year-old healthy male patient was diagnosed with chronic apical periodontitis of his lower left first molar, complaining of some pain in the past and persistent minor discomfort. Tooth was restored with mesioocclusodistal composite filling. Patient reported no trauma or orthodontic treatment in the past. Radiograph (Figure 1) revealed substantial interradicular periapical pathology extending to both roots and an external inflammatory root resorption in the apical third of the mesial root. Lesion was rather irregular in shape but with well-defined apical radiolucency. Shortened root was a sign of a more advanced case. Patient agreed to proceed with proposed endodontic treatment. In addition to the biomechanical preparation of the root canal, calcium hydroxide was used as a temporary therapeutic agent for a period of three months (Figure 2). After the period of calcium hydroxide treatment, thermoplastic gutta-percha obturation was performed. Clinical significance of external root resorption results mainly from the fact that the process perforates radicular lumen. The physiological foramen and the anatomical apex become indistinct. This fact needs to be respected when establishing the definitive working length. Follow-up radiograph was obtained two months after the permanent obturation, showing satisfactory permanent root canal filling (Figure 3). Patient reported minor discomfort that lasted for two or three days following the procedure. All the symptoms had diminished completely at the time of follow-up examination.

### 2.2. Case 2

**Figure 4:**
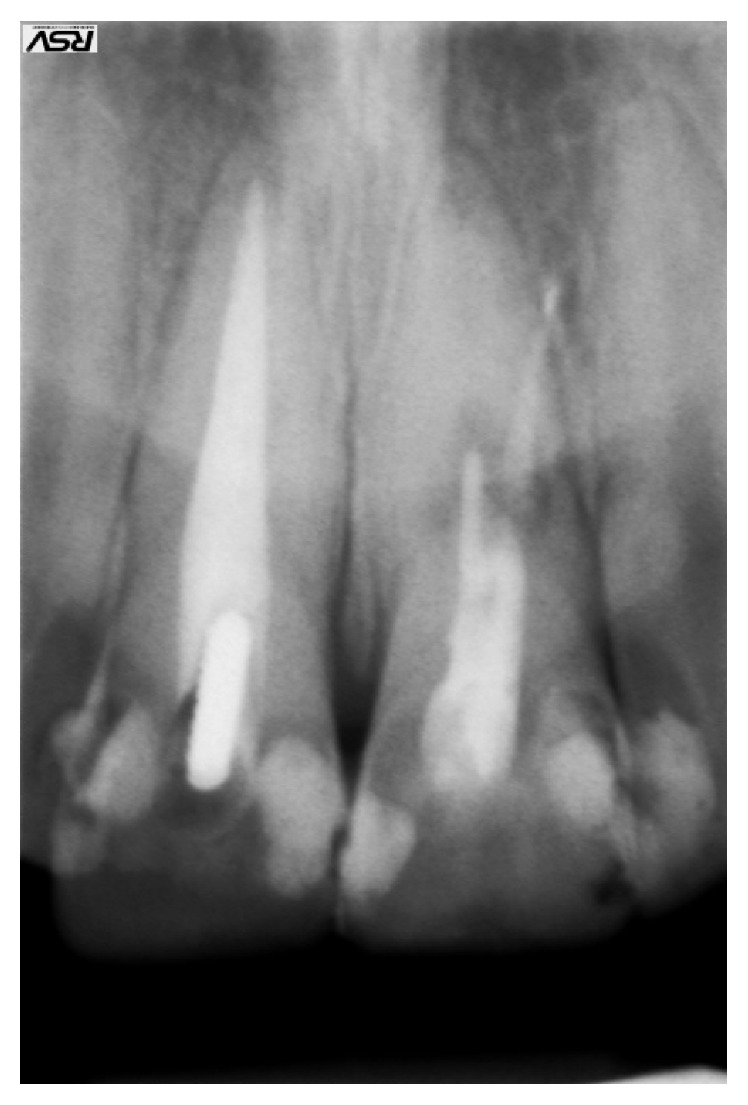
Initial radiograph.

**Figure 5:**
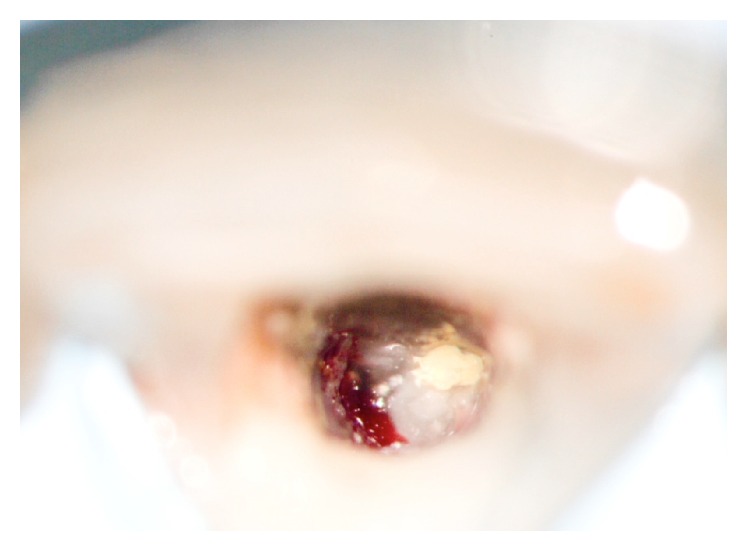
Access cavity with granuloma (left) and failed root filling (right).

An 18-year-old healthy female patient, initially diagnosed with irreversible acute pulpitis, was referred to our practice after endodontic treatment of her upper central incisor affected by internal resorption had failed. Only the coronary root canal was filled. Radiograph revealed that there was an accidental root perforation present and apical section of the root canal remained unfilled (Figure 4). Internal resorption lacuna was visible in the middle third of the root. Patient's informed consent was obtained prior to endodontic retreatment explaining the rationale for treatment and possible alternatives. The basic requirement in the management of this case was the total removal of resorption granuloma. The procedure was performed under an operating microscope. Access cavity provided a view of the residual resorption granuloma that spontaneously perforated into the periodontal crevice together with failed root canal filling (Figure 5).

**Figure 6:**
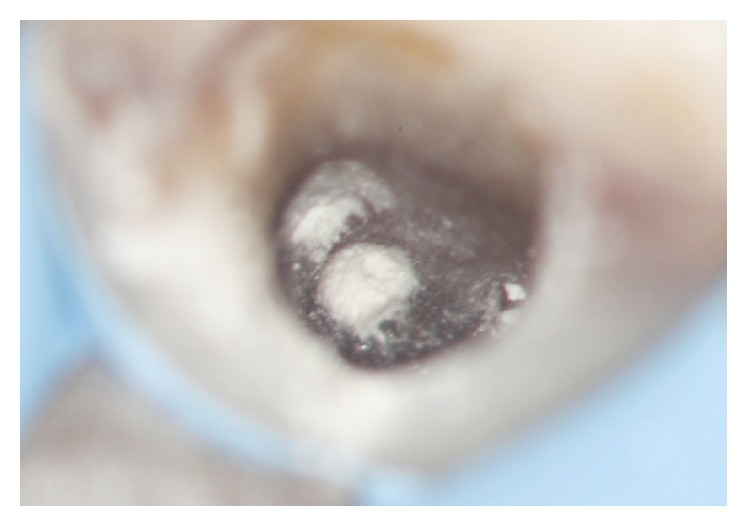
Both perforations covered with MTA.

**Figure 7:**
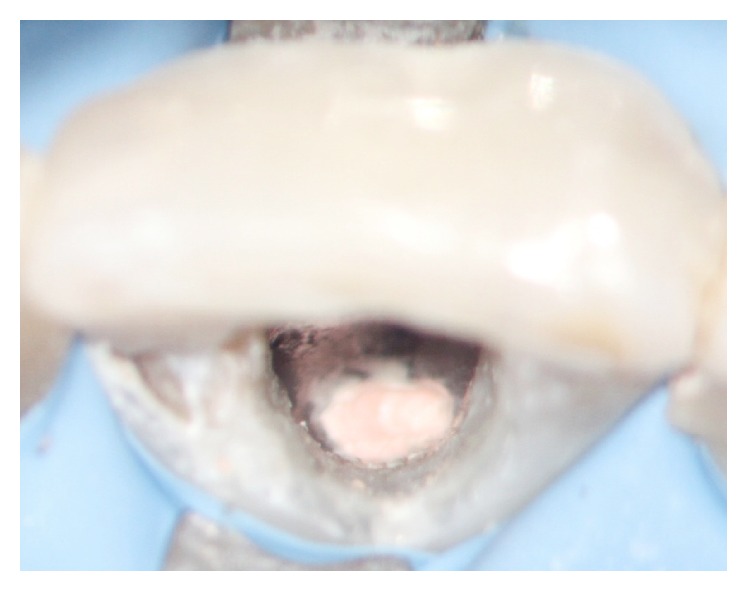
Access cavity with permanent root filling in place.

**Figure 8:**
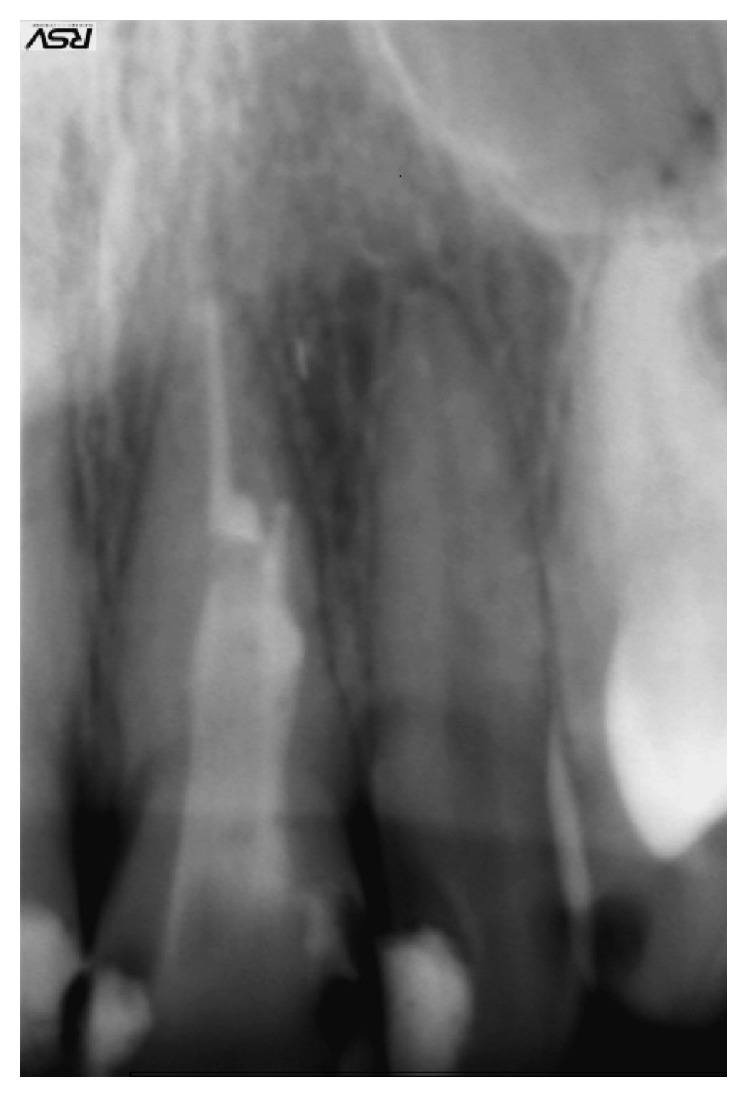
Postoperative radiograph.

**Figure 9:**
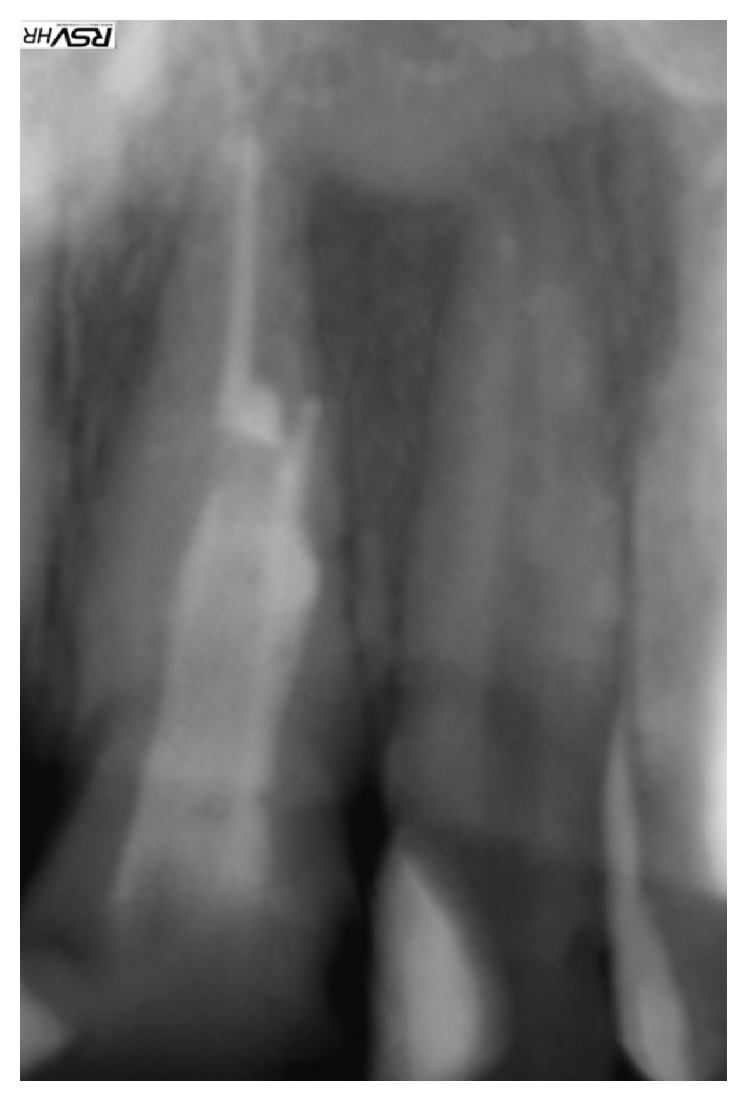
Radiograph at 1-year follow-up.

Similarly to other types of resorption, calcium hydroxide was used as an intracanal medicament for three months. After the calcium hydroxide treatment was completed, both perforations (granuloma and root perforation) were covered with mineral trioxide aggregate (MTA) material (Figure 6) and thermoplastic gutta-percha was used as a permanent root canal filling (Figure 7). Root canal, resorption cavity, and root perforation were filled successfully. We used fiber-reinforced composite post to mechanically strengthen hard dental tissues (Figure 8). Radiograph at one-year follow-up examination showed adequate healing process in the periapical area with new bone formation (Figure 9). Patient reported no subjective complaints regarding the tooth.

## 3. Discussion

Laux et al. conducted a study that associated clinical finding of root resorption with the histological examination [21]. In the study 18% of resorption cases were detected radiographically while the histological examination identified up to 80% of cases. Only resorption that manifested itself via the shortened root was diagnosed reliably. Periapical inflammation is often discussed as possible cause of a radicular external resorption. The severity of resorption is proportional to the duration of the periapical inflammation. Histological studies show that the external resorption of cementum and dentin is due to the activity of the granulation tissue in the area of chronic inflammatory process [22, 23]. We can conclude that periapical lesions such as granulomas and cysts may coexist with the apical external root resorption. These resorptions may not even be visible radiographically. Several authors claimed that the use of endodontic microscope may be beneficial, especially when managing more difficult cases [24, 25]. Schwarze et al. [26] stated that most of the accessory mesiobuccal canals in maxillary molars can only be identified via operating microscope. The vast majority of published papers supporting these views are mostly case reports or small sample studies. On the other hand, Del Fabbro et al. [27] conducted a Cochrane systematic review study in this field. The study found no evidence that allowed them to assess whether magnification improves the success rate of endodontic treatment. There is a need for further research by means of randomized controlled trials. In the view of current scientific evidence, root resorption occurs quite regularly in the daily endodontic practice. However, there is little or no evidence in the current literature with regard to the success rate of root resorption treatment. Our main advice for management of the root resorption is directly related to the expected clinical outcome; resorption process that persists following endodontic treatment is a clear indication for the retreatment. We need to consider the possibility that physiological foramen may have been transposed up to the anatomical apex. Prognosis of root resorption treatment is directly influenced by the quality of endodontic treatment. Cvek [28] reported 96% success rate utilizing the treatment protocol of calcium hydroxide treatment followed by permanent gutta-percha obturation. Wide open apical foramen is more difficult to manage and the root canal may often overfill. Total removal of a resorption granuloma, the use of calcium hydroxide treatment, and adequate sealing of a permanent root canal filling are paramount for achieving long-term success.
